# Microbial Communities in a Serpentinizing Aquifer Are Assembled through Strong Concurrent Dispersal Limitation and Selection

**DOI:** 10.1128/mSystems.00300-21

**Published:** 2021-09-14

**Authors:** Lindsay I. Putman, Mary C. Sabuda, William J. Brazelton, Michael D. Kubo, Tori M. Hoehler, Tom M. McCollom, Dawn Cardace, Matthew O. Schrenk

**Affiliations:** a Department of Earth and Environmental Sciences, Michigan State University, East Lansing, Michigan, USA; b Department of Microbiology and Molecular Genetics, Michigan State University, East Lansing, Michigan, USA; c Department of Earth and Environmental Sciences, University of Minnesota—Twin Cities, Minneapolis, Minnesota, USA; d BioTechnology Institute, University of Minnesota—Twin Cities, St. Paul, Minnesota, USA; e Department of Biology, University of Utah, Salt Lake City, Utah, USA; f SETI Institute, Mountain View, California, USA; g Exobiology Branch, NASA Ames Research Center, Moffett Field, California, USA; h Laboratory for Atmospheric and Space Physics, UCB 600, University of Colorado—Boulder, Boulder, Colorado, USA; i Department of Geosciences, University of Rhode Island, Kingston, Rhode Island, USA; University of Illinois at Urbana-Champaign

**Keywords:** serpentinization, groundwater, community assembly, extreme pH, microbial ecology, deep subsurface, hard-rock aquifer

## Abstract

In recent years, our appreciation of the extent of habitable environments in Earth’s subsurface has greatly expanded, as has our understanding of the biodiversity contained within. Most studies have relied on single sampling points, rather than considering the long-term dynamics of subsurface environments and their microbial populations. One such habitat are aquifers associated with the aqueous alteration of ultramafic rocks through a process known as serpentinization. Ecological modeling performed on a multiyear time series of microbiology, hydrology, and geochemistry in an ultrabasic aquifer within the Coast Range Ophiolite reveals that community assembly is governed by undominated assembly (i.e., neither stochastic [random] nor deterministic [selective] processes alone govern assembly). Controls on community assembly were further assessed by characterizing aquifer hydrogeology and microbial community adaptations to the environment. These analyses show that low permeability rocks in the aquifer restrict the transmission of microbial populations between closely situated wells. Alpha and beta diversity measures and metagenomic and metatranscriptomic data from microbial communities indicate that high pH and low dissolved inorganic carbon levels impose strong environmental selection on microbial communities within individual wells. Here, we find that the interaction between strong selection imposed by extreme pH and enhanced ecological drift due to dispersal limitation imposed by slow fluid flow results in the undominated assembly signal observed throughout the site. Strong environmental selection paired with extremely low dispersal in the subsurface results in low diversity microbial communities that are well adapted to extreme pH conditions and subject to enhanced stochasticity introduced by ecological drift over time.

**IMPORTANCE** Microbial communities existing under extreme or stressful conditions have long been thought to be structured primarily by deterministic processes. The application of macroecology theory and modeling to microbial communities in recent years has spurred assessment of assembly processes in microbial communities, revealing that both stochastic and deterministic processes are at play to different extents within natural environments. We show that low diversity microbial communities in a hard-rock serpentinizing aquifer are assembled under the influence of strong selective processes imposed by high pH and enhanced ecological drift that occurs as the result of dispersal limitation due to the slow movement of water in the low permeability aquifer. This study demonstrates the important roles that both selection and dispersal limitation play in terrestrial serpentinites, where extreme pH assembles a microbial metacommunity well adapted to alkaline conditions and dispersal limitation drives compositional differences in microbial community composition between local communities in the subsurface.

## INTRODUCTION

Recent estimates of the distribution of biomass on Earth indicate that bacteria are the second largest reservoir of biomass (∼70 gigatons of carbon) and that the majority of bacterial and archaeal biomass is hosted within subsurface environments (∼64 gigatons of carbon) (1). In the last decade, several deep subsurface environments have been studied and characterized in detail, allowing us to improve our understanding of the composition and function of microbial communities in this biome and the important roles they play in global biogeochemical cycling (2–4). One type of deep subsurface habitat that has become the subject of intensive study over the last decade is serpentinized peridotite. Serpentinization is a geochemical reaction that hydrates ultramafic rock (peridotite) (5) and generates high pH, reducing fluids with abundant hydrogen (6). In recent years, the microbiology of serpentinizing systems has been studied in a variety of marine (7–9) and terrestrial (6, 10–16) environments. This work has confirmed the presence of endemic, low diversity, microbial communities capable of tolerating the extreme pH (15, 16) and utilizing available hydrogen (17, 18), carbon monoxide (17, 19), methane (14, 20), acetate (6, 14, 21), formate (18–21), and sulfur compounds (22) for both dissimilatory and assimilatory metabolic processes. Combined, this work has provided ample evidence that distinct communities of microorganisms inhabit serpentinizing environments and that they play active roles in these ecosystems.

To date, the ecological forces that structure microbial communities and drive turnover within and between microbial communities in serpentinizing systems have yet to be investigated. Community assembly describes the stochastic (dispersal and ecological drift) and deterministic (environmental and/or biological selection) ecological processes that structure observed microbial communities (23, 24). Advances in DNA sequencing technology in the last 2 decades (25), paired with the more recent implementation of community assembly theory and modeling of sequencing data from microbial ecosystems, have provided new insights into the mechanisms that structure microbial communities in a variety of conditions (24, 26–36). Assessments of community assembly in groundwater systems have shown that selection and dispersal processes play an extremely important role in structuring both the environmental resource landscape as well as microbial communities (29, 31, 32, 34, 37). Due to accessibility, much of this work has been performed in neutral pH, near surface, groundwaters with connectivity to the surface (31, 32, 37). Relatively little work has been done in isolated hard-rock aquifers within the deep subsurface (29, 34). Old groundwaters (>100 years), which are often hosted within hard-rock aquifers in the deep subsurface, are a substantial reservoir of globally available freshwater (38). As these systems are likely dispersal limited, it is important that we understand the ecological forces that structure microbial communities in hard-rock aquifers (29, 34) to better account for the controls on microbial community composition and function within these large reservoirs of valuable water resources (39).

In this study, we apply a null modeling ecological framework (28) that uses the β-nearest taxon index (βNTI) and the abundance-weighted Raup-Crick (RCbray) metric to quantify microbial community assembly processes in an isolated serpentinizing aquifer. We apply the modeling framework to the longest running and highest resolution time series data set collected from a serpentinizing system thus far, to better understand the forces that drive assembly and microbial community turnover within these unique systems. Additionally, we integrate an aqueous geochemistry data set collected alongside microbial samples to better understand environmental drivers of microbial community composition. We perform basic hydrologic modeling to estimate aquifer hydraulic properties to better understand movement of water and the physical environment that microbial communities experience in the subsurface. Our data show that pH imposes a strong selective force on microbial community composition but that, overall, slow fluid flow and poor connectivity in the subsurface isolates microbial communities from one another. This isolation introduces a significant amount of stochasticity by enhancing ecological drift within and between these alkaliphilic microbial communities.

## RESULTS

### Geochemistry.

**FIG 1:**
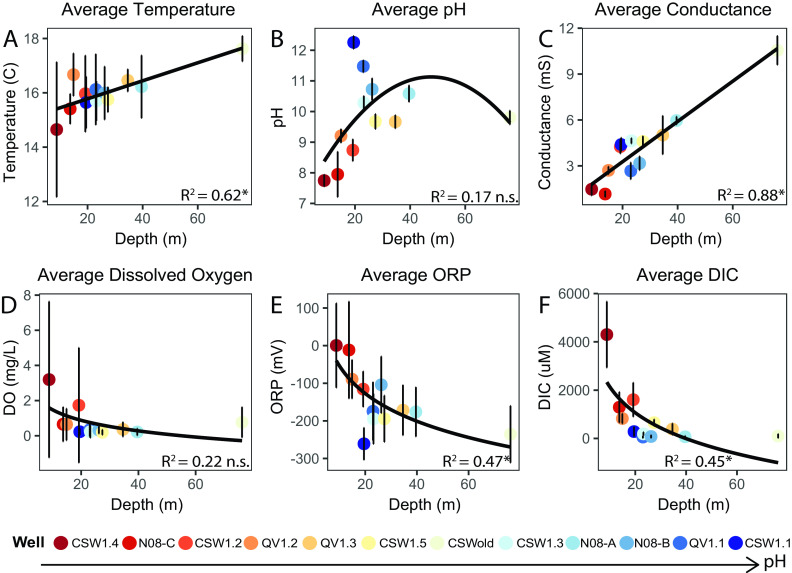
Average measurements for temperature (A), pH (B), specific conductance (C), dissolved oxygen (DO) (D), oxidation reduction potential (ORP) (E), and dissolved inorganic carbon (DIC) (F) in each well plotted against depth. Averages were calculated using data from 2011 to 2017. *R*^2^ coefficients from regression models are listed in the bottom right corner of each plot, where an asterisk indicates a significant correlation (*P* ≤ 0.05) and n.s. indicates a nonsignificant correlation. Wells are organized from lowest to highest average pH in the legend.

Fluids were sampled from 12 wells of various depths (7 to 76 m) that access a high pH aquifer in the serpentinizing ophiolite at the Coast Range Ophiolite Microbial Observatory (CROMO) in Northern California (40). Fluids were sampled two or three times per year over the course of 6 years. Sampled fluids, which are pumped under positive pressure via permanently installed submersible pumps, traveled directly through a chamber with a multiprobe meter to measure temperature, pH, dissolved oxygen (DO), oxidation-reduction potential (ORP), and specific conductance (11, 15). Luer-lock syringes were attached directly to the pump outflow tubing for anoxic sampling of fluids to support subsequent chemical analyses as described in Text S1 in the supplemental material. Analysis of the fluid chemistry (see Table S1 at https://doi.org/10.6084/m9.figshare.14983851) over the duration of the study shows that CROMO fluids generally become more reducing, increase in temperature, salinity, and pH, and decrease in DO and dissolved inorganic carbon (DIC) concentrations with depth (Fig. 1A to F).

10.1128/mSystems.00300-21.1 TEXT S1 Additional Materials and Methods text for sample collection, well head GPS measurements, topographic profile and cross section construction, estimation of aquifer properties, tritium analyses, DNA and RNA extraction, and the ecological modeling framework. Additional discussion of homogenizing dispersal in partially cased wells and the use of distance-based greedy clustering in 16S rRNA sequence processing are also included. Download Text S1, DOCX file, 0.09 MB. Copyright © 2021 Putman et al. 2021 Putman et al. https://creativecommons.org/licenses/by/4.0/ This content is distributed under the terms of the Creative Commons Attribution 4.0 International license.

### Ecological modeling.

DNA and RNA were extracted from microbial communities that were captured on 0.22-μm Sterivex filter cartridges (Millipore, Billerica, MA, USA) during sampling trips from 2011 to 2017 as described below and in Text S1. The following diversity and ecological modeling results are based on a data set generated from gene amplicon sequencing of the V4 region of the 16S rRNA gene. The data set utilized for these analyses consists of 104 samples, 5,974,056 sequence reads, and 13,444 operational taxonomic units (OTUs) clustered at a 3% distance threshold as described below. The 16S rRNA gene amplicon data set represents 51 different phyla, and community composition is largely dominated by members of the *Betaproteobacteriales* and *Clostridiales* orders (see Fig. S1 in the supplemental material). The OTU count table (CROMO_Filtered_FINAL_counts.xlsx) utilized for the following analyses and ecological modeling are published on Figshare (https://figshare.com/projects/Community_Assembly_in_Serpentinizing_Ophiolites/101648).

10.1128/mSystems.00300-21.2 FIG S1 Stacked bar plot showing average relative abundance of each taxonomic order represented in the 50 most abundant OTUs in the data set. Normalized community composition was averaged across all samples for an individual well to determine the “general” community composition within each well. Wells are organized from lowest to highest average pH. Download FIG S1, EPS file, 1.0 MB. Copyright © 2021 Putman et al. 2021 Putman et al. https://creativecommons.org/licenses/by/4.0/ This content is distributed under the terms of the Creative Commons Attribution 4.0 International license.

Ecological modeling of the microbial communities was carried out to characterize the relative contributions of different community assembly processes, using the framework developed by Stegen and colleagues (28). This framework employs null modeling techniques to generate randomized communities that can be compared with observed microbial community composition to determine whether microbial communities are more or less similar to each other than would be expected if communities assembled by random chance. First, βNTI values are calculated to differentiate between deterministic (selective) and stochastic (random) processes. Significant βNTI values indicate that deterministic processes are responsible for observed differences between microbial communities in a given pairwise comparison (37). Nonsignificant βNTI values indicate that stochastic processes are responsible for observed differences between microbial communities (28). Stochastic assembly processes are further investigated using the RCbray metric to characterize the roles of dispersal and ecological drift (28). A detailed description of each community assembly process associated with ecological modeling results (28) can be found in Table 1. βNTI (CROMO_weighted_bNTI_matrix.csv) and RCbray (CROMO_RCbray_matrix.xlsx) matrices used for the following analyses are available on Figshare (https://figshare.com/projects/Community_Assembly_in_Serpentinizing_Ophiolites/101648).

**TABLE 1:** Description of community assembly processes associated with ecological modeling results

Process	Description	Deterministic or stochastic	Model result
Variable selection	Communities are more different from each other than can be expected by random chance (i.e., different physical/chemical conditions in samples drive differing community composition).	Deterministic	βNTI > 2
Homogeneous selection	Communities are more similar to each other than can be expected by random chance (i.e., similar physical/chemical conditions in samples drive community composition to be similar).	Deterministic	βNTI < −2
Dispersal limitation	Microbial communities between samples are unable to interact due to separation by space or time. When communities cannot interact, variation in composition caused by ecological drift over time results in communities that are less similar to each other than expected by chance.	Stochastic	|βNTI| < 2 and RCbray > 0.95
Homogenizing dispersal	Microbial communities between samples can freely interact. Free and easy mixing between communities results in communities that are more similar to each other than expected by chance.	Stochastic	|βNTI| < 2 and RCbray < −0.95
Undominated	While both deterministic and stochastic processes are at play, differences in observed community composition between samples cannot be explained by either selection or random processes (i.e., nonsignificant βNTI and RCbray result).	Both	|βNTI| < 2 and |RCbray| < 0.95

**FIG 2:**
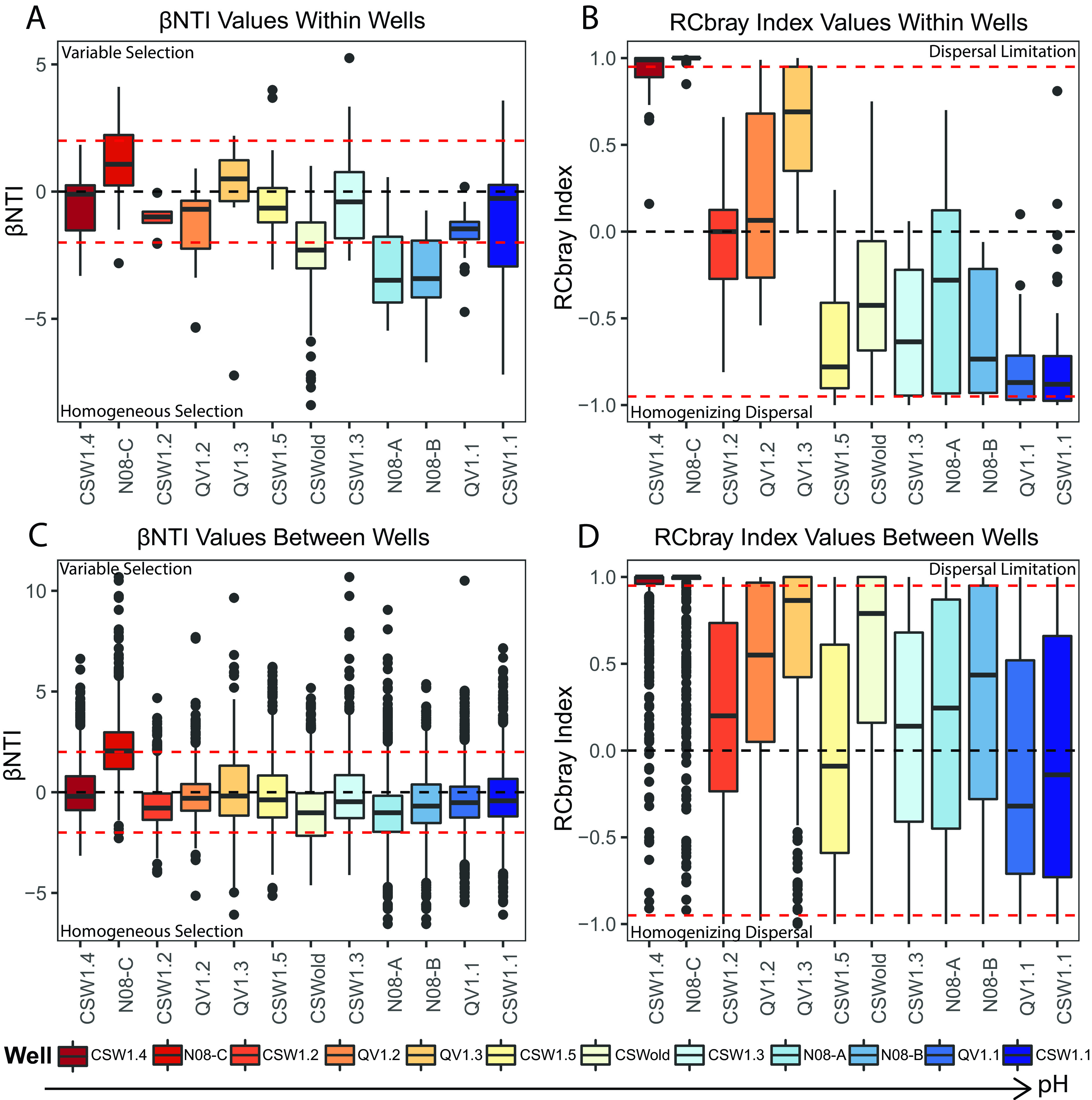
Boxplots showing the distribution of βNTI (A) and RCbray (B) values within individual wells over time and the distribution of βNTI (C) and RCbray (D) values between wells over time. Boxplots represent pairwise comparisons within individual wells over time (A and B), and pairwise comparisons between samples from each individual well and all other wells (C and D). The dashed black line in plots represents the null expectation. Dashed red lines in each plot represent significance thresholds for both the βNTI and RCbray metrics. Plotted RCbray values (B and D) do not include values from pairwise comparisons that have significant βNTI values. See Table 1 for further clarification and interpretation of model results. Wells are organized from lowest to highest average pH in boxplots. Boxplots display the distribution of the data, where the main colored box displays the interquartile range (25th to 75th percentile) of the data. Solid lines within the boxes represent the median of the data. Whiskers on the plot display the maximum and minimum expected values of the data distribution, and black colored points represent outlier data points with respect to the plotted distribution.

Community assembly processes were assessed within individual wells over time (Fig. 2A and B) and between wells over time (Fig. 2C and D). Within individual wells, selection processes do play an important role and can account for 9 to 66% of microbial community turnover observed between samples as a function of time (see Table S2 at https://doi.org/10.6084/m9.figshare.14983857). However, individual wells with more neutral pH conditions (7.5 to 9) display stochastic assembly signatures (|βNTI| < 2), with some influence of variable selection (βNTI > 2) between certain time points (Fig. 2A). The neutral pH well N08-C shows a particularly strong influence of variable selection (25%) over time, indicating that local geochemical conditions may fluctuate enough within the well to drive greater than expected microbial community turnover (see Table S2 at https://doi.org/10.6084/m9.figshare.14983857). Moderate pH (9 to 10.5) wells are primarily influenced by stochastic processes over time with some influence of both variable and homogeneous selection processes between certain time points (Fig. 2A). Extreme pH (10.5 to 12) wells become more strongly influenced by homogeneous selection (βNTI < −2) (Fig. 2A), although stochastic processes still play a significant role within these wells, accounting for 34 to 91% of assembly within an individual well (see Table S2 at https://doi.org/10.6084/m9.figshare.14983857). In general, no single assembly process was able to explain variation in microbial community composition between time points (14 to 84% undominated processes) (Fig. 2B; see Table S2 at https://doi.org/10.6084/m9.figshare.14983857), indicating that no single assembly process can explain variation in microbial community composition between time points (undominated assembly).

In pairwise comparisons between individual wells, βNTI mean and median values lie close to zero, indicating that stochastic processes dominate (Fig. 2C). Nevertheless, selection processes still have some influence on between-well comparisons. Variable selection strongly influences pairwise comparisons between the neutral pH (7.5 to 9) wells CSW1.4 and N08-C with other higher pH (9 to 12) wells at the site, while homogeneous selection plays a stronger role in pairwise comparisons between the deepest wells at the site N08-A (39.6 m) and CSWold (76.2 m) and other medium to deep wells (20 to 35 m) with moderate to extreme pH (9 to 12) (Fig. 2C). Further characterization of stochastic processes at play in comparisons between wells reveals that dispersal limitation plays a larger role compared to within-well comparisons, but that overall, no single assembly process dominates between-well comparisons (undominated assembly) (Fig. 2B and D). Mean and median RCbray values trend more toward dispersal limitation, with numerous samples within the distribution of pairwise comparisons crossing the significance threshold (RCbray > 0.95) (Fig. 2D). A small proportion of between-well pairwise comparisons cross the significance threshold for being under the influence of homogenizing dispersal (RCbray < −0.95), suggesting that microbial communities associated with those specific wells are mixing and interacting with each other (Fig. 2D). These comparisons are primarily associated with the two partially cased wells at the site, CSW1.1 and QV1.1, and nearby wells at similar depths. Discussion of the increased influence of homogenizing dispersal in wells CSW1.1 and QV1.1 (87) can be found in Text S1.

Mantel tests were used to assess correlations between ecological modeling metrics and measured environmental parameters (see Table S3 at https://doi.org/10.6084/m9.figshare.14983860). Environmental variables were transformed into Euclidean distance matrices so they could be correlated with ecological modeling matrices. βNTI values were significantly correlated with pH (*R* = 0.29), ORP (*R* = 0.23), and DIC (*R* = 0.14) (Fig. S2). This correlation indicates that greater differences in environmental measurements of pH, ORP, and DIC are associated with variable selection (i.e., more positive values of βNTI). Samples that are more different from each other with respect to pH, ORP, and DIC conditions are likely to be influenced by variable selection, whereas samples with similar pH, ORP, and DIC conditions are likely to be influenced by homogeneous selection. RCbray values were significantly correlated with well depth (*R* = 0.32), temperature (*R* = 0.15), pH (*R* = 0.27), specific conductance (*R* = 0.34), ORP (*R* = 0.30), and DIC (*R* = 0.33) (Fig. S3). That is, samples with greater differences in environmental conditions are more likely to be influenced by dispersal limitation (high RCbray), while samples with more similar environmental conditions are more likely to show influences of homogenizing dispersal. The relative strength of selection processes on microbial communities seems to be tightly linked to pH, ORP, and DIC conditions, while the influence of stochastic processes is correlated with a wider range of environmental conditions.

10.1128/mSystems.00300-21.3 FIG S2 βNTI plotted as a function of the difference in pH (A), oxidation reduction potential (ORP) (B), and dissolved inorganic carbon (DIC) (C). Solid black lines in plots represent fitted regression trend lines based on significant Mantel test correlations (see Table S3 at https://doi.org/10.6084/m9.figshare.14983860). Horizontal red lines at βNTI values of 2 and −2 represent the significance threshold for the βNTI metric which indicate that homogeneous selection (βNTI < −2) or variable selection (βNTI > 2) govern assembly. Download FIG S2, TIF file, 0.4 MB. Copyright © 2021 Putman et al. 2021 Putman et al. https://creativecommons.org/licenses/by/4.0/ This content is distributed under the terms of the Creative Commons Attribution 4.0 International license.

10.1128/mSystems.00300-21.4 FIG S3 RCbray plotted as a function of the difference in depth (A), temperature (B), pH (C), specific conductance (D), oxidation reduction potential (ORP) (E), and dissolved inorganic carbon (DIC) (F). Solid black lines in plots represent fitted regression trend lines based on significant Mantel test correlations (see Table S3 at https://doi.org/10.6084/m9.figshare.14983860). Horizontal red lines at RCbray values of 0.95 and −0.95 represent the significance threshold for the RCbray metric which indicate that dispersal limitation (RCbray > 0.95) or homogenizing dispersal (RCbray < −0.95) govern assembly. Download FIG S3, TIF file, 0.4 MB. Copyright © 2021 Putman et al. 2021 Putman et al. https://creativecommons.org/licenses/by/4.0/ This content is distributed under the terms of the Creative Commons Attribution 4.0 International license.

**FIG 3:**
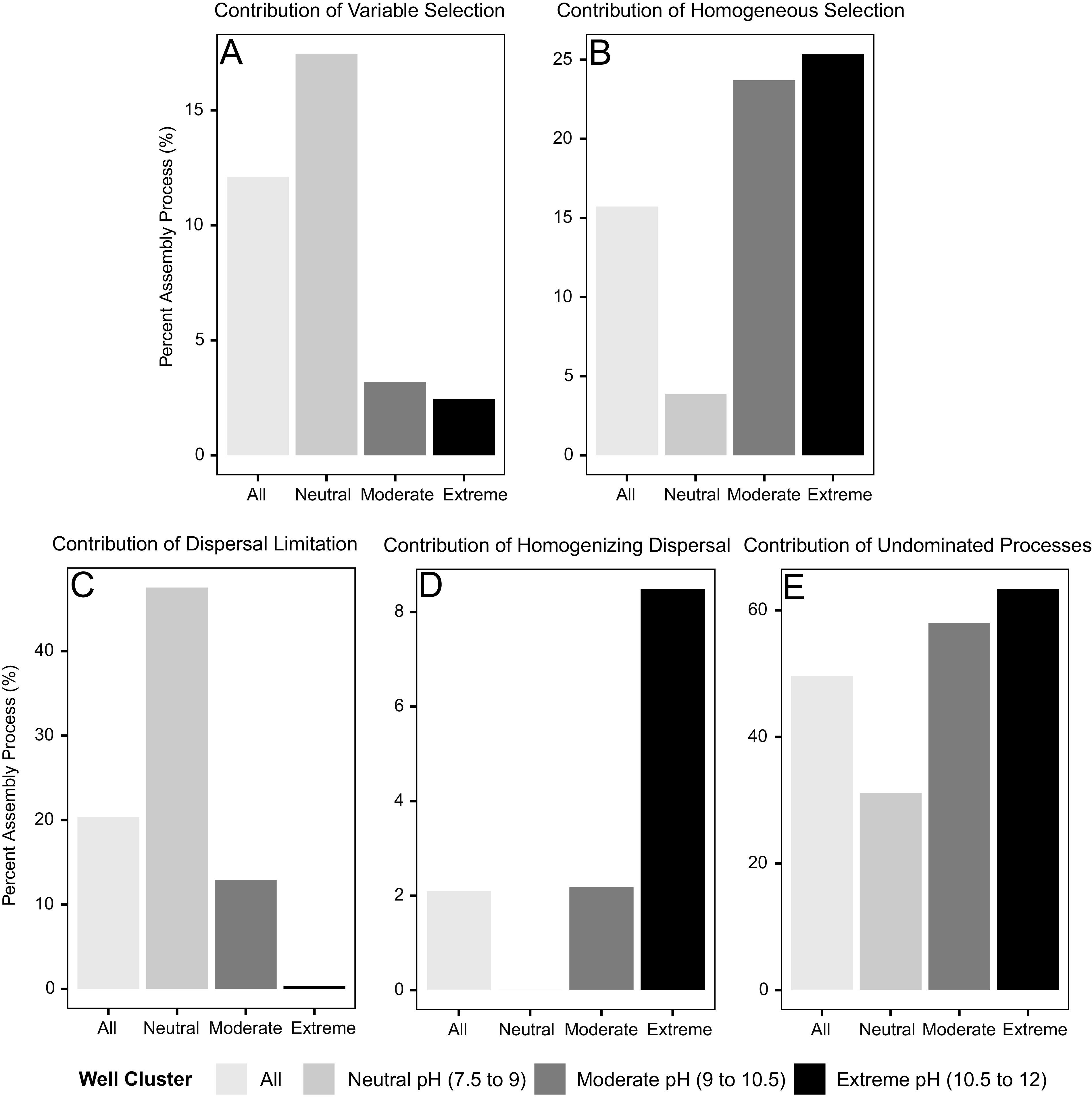
Bar plots showing the overall contribution of variable selection (A), homogeneous selection (B), dispersal limitation (C), homogenizing dispersal (D), and undominated processes (E) for all wells, neutral pH wells (pH 7.5 to 9), moderate pH wells (pH 9 to 10.5), and extreme pH wells (pH 10.5 to 12). The contribution (percentage) of each assembly process was calculated within each grouping of samples by dividing the number of significant pairwise comparisons for each assembly process by the total number of pairwise comparisons within the sample grouping. Pairwise comparisons include within-well comparisons and between-well comparisons for all wells and wells within each pH category.

Finally, the contribution of each community assembly process was quantified by compiling the number of significant pairwise comparisons for each process and dividing these numbers by the total number of pairwise comparisons. Overall, half of the comparisons could not be attributed to a single assembly process (50% undominated processes), with the other half distributed among dispersal limitation (20%), homogeneous selection (16%), variable selection (12%), and homogenizing dispersal (2%) (Fig. 3; see Table S2 at https://doi.org/10.6084/m9.figshare.14983857). Due to the importance of pH in structuring ecological niches in the system (Fig. S4), community assembly processes were also quantified for all pairwise comparisons between neutral pH (7.5 to 9) wells (CSW1.4, N08-C, CSW1.2, and QV1.2), moderate pH (9 to 10.5) wells (QV1.3, CSW1.5, CSWold, and CSW1.3) and extreme pH (10.5 to 12) wells (N08-A, N08-B, QV1.1, and CSW1.1). When separated into discrete pH ranges, trends in the influence of community assembly processes with pH are evident. As pH increases, the role of variable selection decreases (from 17% to 2%), the role of homogeneous selection increases (from 4% to 25%), the role of dispersal limitation decreases (from 48% to 0%), the role of homogenizing dispersal increases (from 0% to 8%), and the role of undominated processes increase (from 31% to 63%) (Fig. 3A to E; see Table S2 at https://doi.org/10.6084/m9.figshare.14983857). The increasing role of homogeneous selection indicates that microbial communities are more like each other than expected by chance as pH conditions become more extreme (Fig. 3B). The shift from dispersal limitation (Fig. 3C) to homogenizing dispersal (Fig. 3D) with increasing pH indicates that microbial communities are less isolated from one another, and interact more, at high pH.

10.1128/mSystems.00300-21.5 FIG S4 Mantel correlogram showing significant phylogenetic signal at short phylogenetic distances. The correlogram plots the phylogenetic distance of each OTU in the data set against the average pH niche value calculated for each OTU. Solid boxes represent significant correlations, and open boxes represent nonsignificant correlations. Significant correlations at short phylogenetic distances indicate that phylogenetic signal occurs at small phylogenetic distances, making it appropriate to assess phylogenetic turnover amongst closely related organisms. Download FIG S4, EPS file, 0.6 MB. Copyright © 2021 Putman et al. 2021 Putman et al. https://creativecommons.org/licenses/by/4.0/ This content is distributed under the terms of the Creative Commons Attribution 4.0 International license.

### Physical controls on microbial dispersal.

Ecological modeling results revealed that CROMO microbial communities are overwhelmingly assembled through undominated processes (50% undominated processes; see Table S2 at https://doi.org/10.6084/m9.figshare.14983857), where neither stochastic nor deterministic processes entirely govern assembly (Table 1). Since the modeling results indicate that half the data analyzed in the model are governed by a mixture of stochastic and deterministic processes that cannot be well defined by the model (Table 1), alternative assessments of dispersal and environmental selection (following section) were performed to better understand physicochemical drivers of assembly at CROMO. Connectivity of the aquifer to the surface and estimation of aquifer hydraulic properties were assessed to better understand dispersal within the subsurface.

Characterization of the CROMO aquifer’s connectivity to the surface and aquifer hydraulic properties was carried out for this study using tritium analyses and pumping tests as described below. Previous work by Ortiz and colleagues (41) indicated that the main aquifer at CROMO is confined. Tritium analyses (see Table S4 at https://doi.org/10.6084/m9.figshare.14983863) performed on samples collected in May of 2017 were below detection (0.8 tritium unit [TU]) in all wells except for a shallow well, N08-C, where tritium was detected at 0.9 TU.

**FIG 4:**
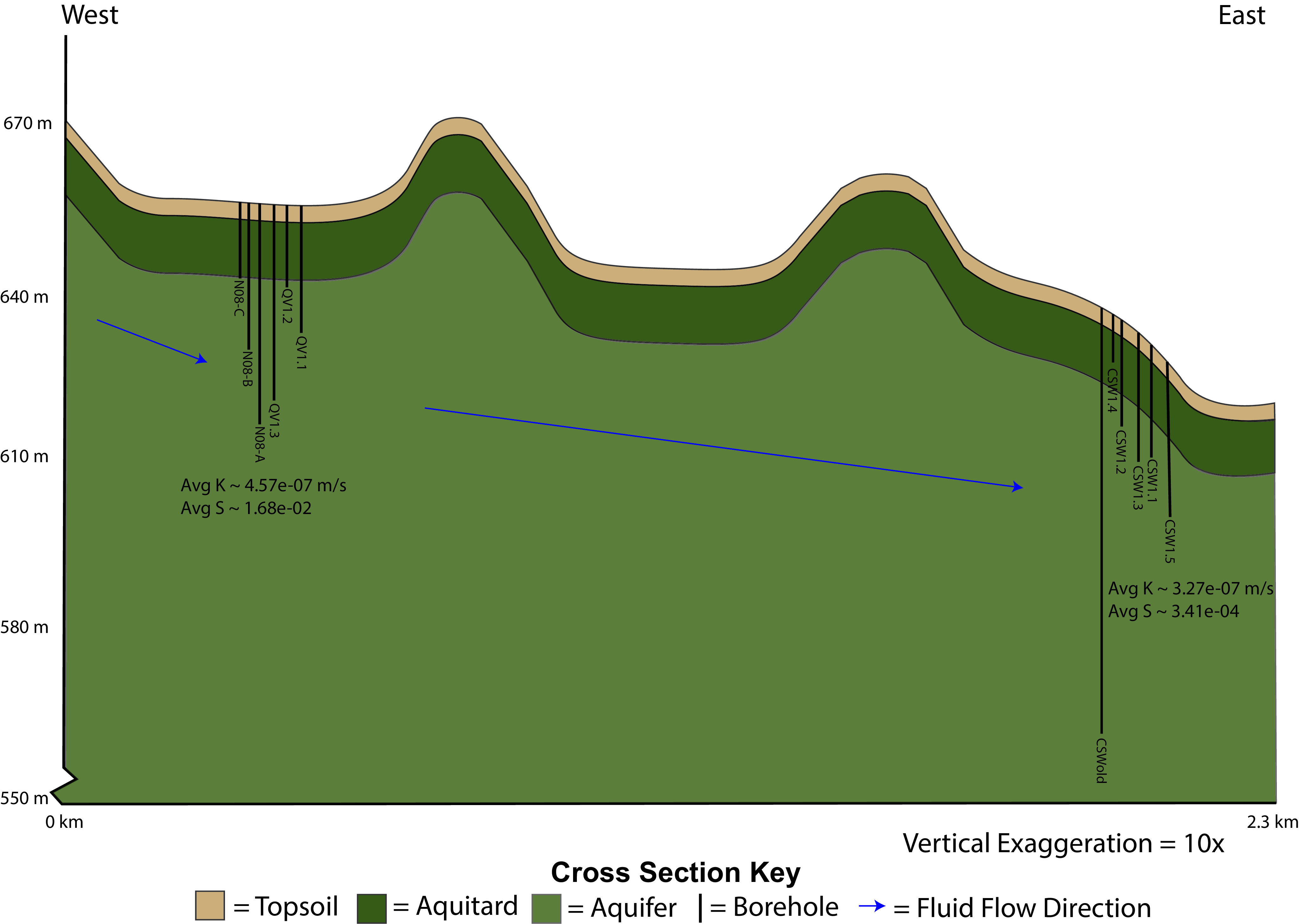
Cross section with 10× vertical exaggeration showing the topographic profile of the landscape around the CROMO wells. Well locations are scattered close to the cross section line, so well locations were estimated in their approximate order from west to east. Wells are drawn to accurate depth based on the surface elevation within the profile. The depths of the topsoil and aquitard layers were estimated based on geophysical mapping done by Ortiz and colleagues (41). Given that the same units were present at both the CSW and QV clusters and were about the same thickness, we made the simplifying assumption that the topsoil and aquitard layers are laterally continuous and of even thickness between well cluster locations. We estimated that the main aquifer was 76.2 m thick. It needed to accommodate the depth of CSWold, but we do not have a good estimate as to the depth where basement rock begins. The general direction of groundwater flow is shown within the diagram, and average estimates for hydraulic conductivity (*K*) and storativity (*S*) at each well cluster are included from Table S5 at https://doi.org/10.6084/m9.figshare.14983866.

Pumping tests used to estimate aquifer hydraulic conductivity (*K*) indicate that water flows through the subsurface at a low rate (*K* = 10^−7^ m/s) (Fig. 4; see Table S5 at https://doi.org/10.6084/m9.figshare.14983866). Estimates of *K* and other hydraulic properties (see Table S5 at https://doi.org/10.6084/m9.figshare.14983866) are in line with estimates obtained at the Samail ophiolite, Sultanate of Oman (42), and Koniambo massif in New Caledonia (43).

### Adaptations to environmental conditions.

Given the distinct geochemical trends observed at CROMO (Fig. 1), a permutational multivariate analysis of variance (PERMANOVA) analysis was performed on Bray-Curtis dissimilarities and measured environmental data to determine how much variance in the community dissimilarity data can be explained by measured environmental data (Table 2). Results from this analysis show that well location (i.e., corresponding subsurface location) (*R*^2^ = 0.468) is the primary driver of differences in microbial community composition. Significant differences in microbial community composition are also seen over time (*R*^2^ = 0.029) and are driven by differences in DIC (*R*^2^ = 0.012), DO (*R*^2^ = 0.011), and pH (*R*^2^ = 0.010) to a lesser extent (Table 2). PERMANOVA results also indicate that a significant amount of variation in microbial community composition is unexplained (residuals *R*^2^ = 0.44) (Table 2). It is important to note that community composition changes related to changes in time, DIC, DO, and pH may already be accounted for as a portion of the large amount of variance explained by individual well location. This observation could explain why the amount of variation described by each individual variable is quite low (*R*^2^ of 0.01 to 0.029). We performed an additional PERMANOVA analysis without the well location variable to further explore this result. While less of the overall variance in community composition is explained (residuals *R*^2^ = 0.69), more variance in community composition is associated with geochemical metadata, especially pH (*R*^2^=0.13) (see Table S6 at https://doi.org/10.6084/m9.figshare.14983869). The increased variance accounted for in the data when considering the well location variable likely includes both measured and unmeasured physicochemical data specific to each well location (Table 2).

**TABLE 2:** Results from PERMANOVA analysis on Bray-Curtis dissimilarities and associated sample metadata

Variable	PERMANOVA results					
	Degrees of freedom	Sum of squares	Mean squares	*F* score	*R* ^2^ ^*a*^	*P* value^*b*^
Well	11	16.239	1.47626	8.0442	0.46783	**0.001**
Day	1	1.018	1.01783	5.5461	0.02932	**0.001**
Temp (°C)	1	0.145	0.14524	0.7914	0.00418	0.702
pH	1	0.355	0.35524	1.9357	0.01023	**0.028**
Conductance (mS)	1	0.274	0.27357	1.4907	0.00788	0.113
DO (mg/liter)	1	0.376	0.37644	2.0512	0.01084	**0.014**
ORP (mV)	1	0.274	0.27357	1.4907	0.00788	0.09
DIC (μM)	1	0.431	0.43122	2.3497	0.01242	**0.006**
Residuals	85	15.599	0.18352		0.4494	
Total	103	34.711			1	

a *R*^2^ values indicate the percent variation in community dissimilarity that can be described by an individual variable.

b Significant *P* values are bolded.

Since pH appears to play an important role in structuring ecological niches at CROMO (Fig. S4; Table 2; see Table S6 at https://doi.org/10.6084/m9.figshare.14983869), as well as microbial communities in a variety of environments (24, 33), the influence of pH in structuring CROMO microbial communities was further assessed. While DO and DIC concentrations were also found to drive changes in community composition (Table 2), DO was not found to be significantly correlated with ecological model βNTI values, which assess the role of environmental selection (Fig. S2; see Table S3 at https://doi.org/10.6084/m9.figshare.14983860). DIC was significantly correlated with βNTI values but is not assessed here, since pH has been shown to govern the amount of available DIC in serpentinizing systems (44). Due to this, environmental selection due to changes in DIC availability should ultimately be controlled by pH conditions.

**FIG 5:**
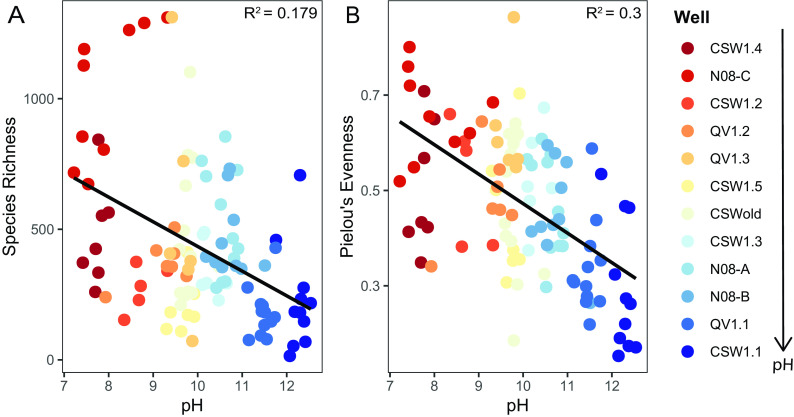
Microbial community richness (A) and evenness (B) plotted against pH. Points represent individual microbial community samples, which are colored by well location. Wells are organized from lowest to highest average pH in the legend. Linear regression lines are plotted in black. Significant (*P* ≤ 0.05) regression *R*^2^ values are displayed in the upper right corner of plots.

**FIG 6:**
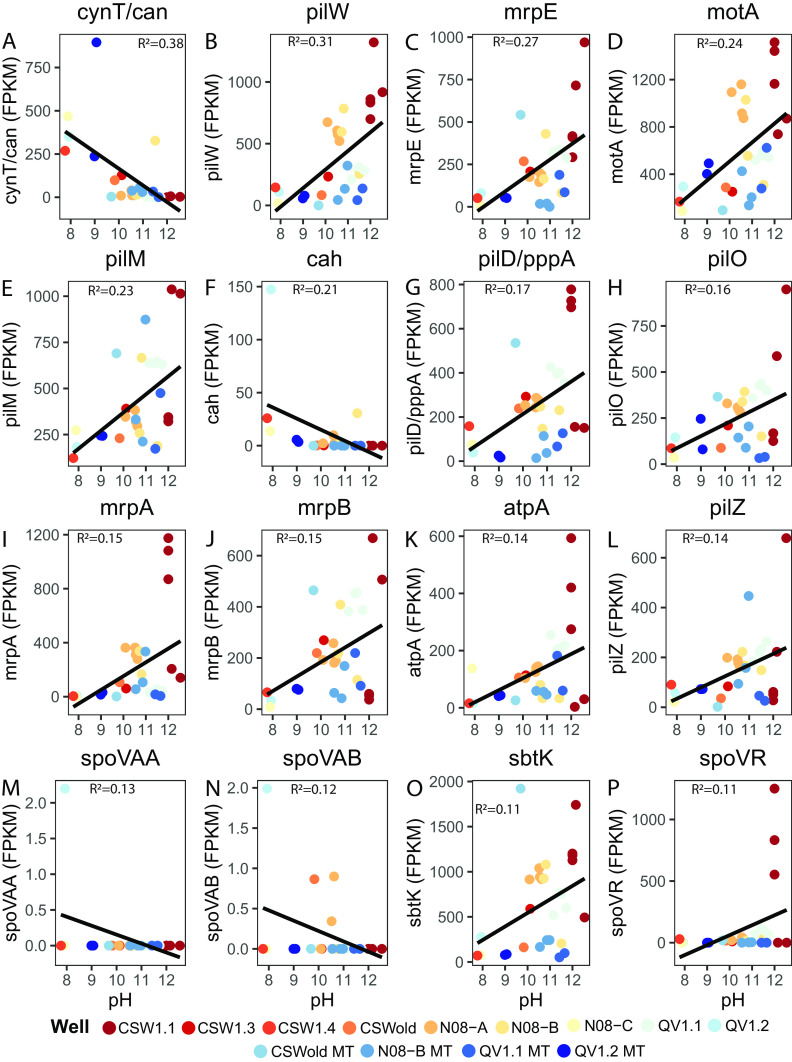
Significant linear regression plots of the normalized abundance of metagenomic and metranscriptomic data in fragments per kilobase per million reads (FPKM) for the genes *cynT*/*can* (A), *pilW* (B), *mrpE* (C), *motA* (D), *pilM* (E), *cah* (F), *pilD*/*pppA* (G), *pilO* (H), *mrpA* (I), *mrpB* (J), *atpA* (K), *pilZ* (L), *spoVAA* (M), *spoVAB* (N), *sbtK* (O), and *spoVR* (P) plotted against pH. Solid black lines represent a linear trend line. Linear regression *R*^2^ values are included at the top of each plot. Samples of metagenomic and metatranscriptomic data are colored by well location. Metatranscriptomic samples are labeled with an MT following the well name in the legend. Functions associated with genes are as follows: motility/adhesion (*pilW*, *motA*, *pilM*, *pilD*/*pppA*, *pilO*, and *pilZ*), Na^+^/H^+^ antiporters (*mrpE*, *mrpA*, and *mrpB*), sporulation (*spoVAA*, *spoVAB*, and *spoVR*), Na^+^-dependent bicarbonate transporters (*sbtK*), Na^+^-dependent V-type ATPase (*atpA*), carbonic anhydrase (*cynT*/*can* and *cah*).

Community richness (number of unique taxa in a community) and evenness (how close in numerical abundance taxa in a community are; measured by Pielou’s evenness index) decrease as pH increases (Fig. 5A and B). This result supports previous reports that pH is a major driver of microbial community diversity at the site (15), as has been seen in numerous other environments (24, 33). To further assess this point, metagenomic and metatranscriptomic data collected during the 2011 to 2013 field campaigns and again in 2016 were screened for genes associated with adaptations to high pH (see Table S7 at https://doi.org/10.6084/m9.figshare.14372030). It is known through physiological studies of alkaliphilic cultures at high pH that a proton (H^+^) gradient exists from cell cytoplasm to cell exterior that can drive loss of protons from the cell (45). To combat this issue, microorganisms must actively transport protons back into the cell (45). The use of cation/proton antiporters, which can transport sodium (Na^+^) or potassium (K^+^) ions out of the cell while transporting protons into the cell, as well as Na^+^-pumping V-type ATPases, which pump Na^+^ ions across the cell membrane to generate ATP, are common mechanisms used by microorganisms growing in alkaline pH conditions (45, 46). We find that CROMO metagenomes have abundant Na^+^/H^+^ and K^+^/H^+^ antiporters, and Na^+^-pumping V-type ATPases (see Table S7 at https://doi.org/10.6084/m9.figshare.14372030). The abundance and transcription of genes encoding carbonic anhydrases *cynT*/*can* and *cah* and sporulation genes *spoVAA* and *spoVAB* decrease with increasing pH (Fig. 6A, F, M, and N), while the abundance and transcription of genes associated with motility, pili for motility and adhesion, Na^+^/H^+^ antiporters, Na^+^-dependent V-type ATPases, Na^+^-dependent bicarbonate transporters, and the sporulation gene *spoVR* increase with increasing pH (Fig. 6B to E, G to L, and O and P). The abundance and transcription of genes associated with motility and adhesion, Na^+^/H^+^ antiporters, Na^+^-dependent bicarbonate transporters, Na^+^-pumping V-type ATPases, sporulation, and survival-related transcription factors increase with increasing salinity and depth (Fig. S5A to R). Despite a great abundance of sporulation genes observed in wells deep (>27 m) within the aquifer, poor transcription of these genes paired with evidence for the transcription of other maintenance and metabolic genes (20, 22) indicate that microbial community members were not actively employing this strategy to cope with extreme environmental conditions at the time of sampling. The abundance and transcription of genes associated with sporulation, Na^+^/H^+^ antiporters, and Na^+^-pumping V-type ATPases decrease with increasing ORP (Fig. S6A to F). The abundance and transcription of genes associated with carbonic anhydrases increase with increasing concentrations of DIC (Fig. S6G and L), while the abundance of genes associated with Na^+^/H^+^ antiporters and with motility and adhesion decrease with increasing concentrations of DIC (Fig. S6H to K). The abundance and transcription of genes associated with motility and adhesion, sporulation, Na^+^-pumping V-type ATPases, Na^+^-dependent bicarbonate transporters, and K^+^/H^+^ symporters increase with increasing temperature (Fig. S6M to V). Linear regression model results for significant and insignificant regressions can be found in Table S8 (https://doi.org/10.6084/m9.figshare.14983872) and Table S9 (https://doi.org/10.6084/m9.figshare.14939202), respectively.

10.1128/mSystems.00300-21.6 FIG S5 Significant linear regression plots of the normalized abundance of metagenomic and metranscriptomic data in fragments per kilobase per million reads (FPKM) for the genes *hspR* (A), *mrpF* (B), *mrpG* (C), *mrpD* (D), *spoVFA* (E), *sbtK* (F), *spoVS* (G), *atpH* (H), and *spoVK* (I) plotted against specific conductance (Cond. [mS]), and genes *hspR* (J), *mrpD* (K), *mrpF* (L), *spoVFA* (M), *spoVS* (N), *atpH* (O), *mrpG* (P), *pilC* (Q), and *spoIVCA* (R) plotted against depth. Solid black lines represent a linear trend line. Linear regression *R*^2^ values are included at the top of each plot. Samples of metagenomic and metatranscriptomic data are colored by well location. Metatranscriptomic samples are labeled with an MT following the well name in the legend. Functions associated with genes are as follows: motility/adhesion (*pilC*), Na^+^/H^+^ antiporters (*mrpF*, *mrpG*, and *mrpD*), sporulation (*spoVFA*, *spoVS*, *spoVK*, and *spoIVCA*), Na^+^-dependent bicarbonate transporters (*sbtK*), Na^+^-dependent V-type ATPase (*atpH*), and survival transcription factors (*hspR*). Download FIG S5, EPS file, 1.5 MB. Copyright © 2021 Putman et al. 2021 Putman et al. https://creativecommons.org/licenses/by/4.0/ This content is distributed under the terms of the Creative Commons Attribution 4.0 International license.

10.1128/mSystems.00300-21.7 FIG S6 Significant linear regression plots of the normalized abundance of metagenomic and metranscriptomic data in fragments per kilobase per million reads (FPKM) for the genes *spoVS* (A), *mrpD* (B), *atpH* (C), *spoVFA* (D), *spoVFB* (E), and *mrpB* (F) plotted against oxidation reduction potential (ORP [mV]), genes *cynT*/*can* (G), *mrpB* (H), *pilM* (I), *mrpG* (J), *mrpC* (K), and *cah* (L) plotted against dissolved inorganic carbon (DIC [μM]), and genes *pilB* (M), *pilQ* (N), *mrpA* (O), *spoVR* (P), *fliD* (Q), *motA* (R), *trkA*/*ktrA* (S), *atpA* (T), *sbtK* (U), and *trkH* (V) plotted against temperature (Temp. [°C]). Solid black lines represent a linear trend line. Linear regression *R*^2^ values are included at the top of each plot. Samples of metagenomic and metatranscriptomic data are colored by well location. Metatranscriptomic samples are labeled with an MT following the well name in the legend. Functions associated with genes are as adhesion/adhesion (*pilM*, *pilB*, *pilQ*, *motA*, and *fliD*), Na^+^/H^+^ antiporters (*mrpD*, *mrpB*, *mrpG*, *mrpC*, *mrpA*), sporulation (*spoVS*, *spoVFA*, *spoVFB*, and *spoVR*), Na^+^-dependent bicarbonate transporters (*sbtK*), Na^+^-dependent V-type ATPase (*atpH* and *atpA*), K^+^/H^+^ symporters (*trkA*/*ktrA* and *trkH*), and carbonic anhydrase (*cynT*/*can* and *cah*). Download FIG S6, EPS file, 1.7 MB. Copyright © 2021 Putman et al. 2021 Putman et al. https://creativecommons.org/licenses/by/4.0/ This content is distributed under the terms of the Creative Commons Attribution 4.0 International license.

In addition to the community diversity, metagenomic, and metatranscriptomic evidence provided, phylogenetic analysis of these communities using the nearest taxon index (a within sample, or alpha diversity, variation of the βNTI metric used for ecological modeling) indicate that CROMO microbial communities are composed of closely related organisms and demonstrate significant phylogenetic clustering (Fig. S7). Phylogenetic clustering indicates that environmental filtering and the conservation of adaptive traits have likely occurred as the result of habitat specialization (47).

10.1128/mSystems.00300-21.8 FIG S7 Nearest taxon index (NTI) plotted within individual wells. NTI values are obtained by comparing observed mean nearest taxon distance (MNTD) values to those generated within a null distribution. The black dashed line represents the null expectation, and the red dashed line indicates the significance threshold. Positive values crossing the significance threshold indicate that microbial communities are phylogenetically clustered. Wells are organized from lowest to highest average pH. Download FIG S7, EPS file, 1.3 MB. Copyright © 2021 Putman et al. 2021 Putman et al. https://creativecommons.org/licenses/by/4.0/ This content is distributed under the terms of the Creative Commons Attribution 4.0 International license.

## DISCUSSION

Given the extreme pH conditions at CROMO, we anticipated that deterministic processes would be dominant, as has been observed in other systems under extreme or stressful environmental conditions (33, 48–52). Ecological modeling results revealed that community assembly at CROMO instead occurs through a combination of deterministic and stochastic processes that cannot be well defined (undominated assembly; see Table S2 at https://doi.org/10.6084/m9.figshare.14983857). Because the model cannot define the stochastic and deterministic processes at work when undominated assembly governs pairwise comparisons, we employed alternative methods to assess the roles of dispersal (Fig. 4; see Table S4 at https://doi.org/10.6084/m9.figshare.14983863) and selection (Fig. 5 and 6 and Fig. S5, S6, and S7; Table 2) within the CROMO aquifer. Here, we explore the combined results and aim to describe the stochastic and deterministic processes at work within the undominated fraction of data identified by the ecological model. We discuss the roles of dispersal and selection in the CROMO aquifer, incorporating results and interpretations from the multiple data streams reported here, as well as previous reports and interpretations of community assembly relevant to observations in this study.

While most assembly processes at CROMO cannot be well defined (undominated assembly), dispersal limitation (20%) and homogeneous selection (16%) are the two primary quantifiable processes at work within the aquifer (Fig. 3; see Table S2 at https://doi.org/10.6084/m9.figshare.14983857). Evidence of dispersal limitation is clear when looking at the hydrogeology of the system (Fig. 4). Tritium levels below detection in well fluids (see Table S4 at https://doi.org/10.6084/m9.figshare.14983863) indicate that the main aquifer is not well connected to the surface and that modern recharge (fluid <50 years in age) does not contribute appreciably to the subsurface reservoir (38, 53, 86). When considered alongside hydraulic conductivity estimates (see Table S5 at https://doi.org/10.6084/m9.figshare.14983866), these results indicate that the CROMO aquifer is a confined system receiving no detectable modern recharge and is characterized by poor connectivity between wells and low rates of fluid flow (Fig. 4). Differences in compositional beta diversity (Bray-Curtis dissimilarity; Table 2) provide additional evidence of dispersal limitation. The mean Bray-Curtis dissimilarity at the site when considering all pairwise comparisons is extremely high (0.8), indicating that compositional diversity of microbial communities have little overlap (54, 55). Differences in well location (and conditions—see results relevant to Table 2 and Table S6 at https://doi.org/10.6084/m9.figshare.14983869) alone account for nearly 50% of observed variation in community dissimilarity, while changes in community composition over time follow at a much lower level of explained variance (∼3%) (Table 2). Additionally, 44% of the variation in microbial community composition could not be explained by environmental variables during PERMANOVA analysis. The unexplained variation in community composition could be the result of stochastic changes in community composition due to ecological drift. Isolation of microbial communities due to dispersal limitation could certainly introduce enough ecological drift within each well location to generate such high levels of microbial community compositional dissimilarity (56–59). Environmental heterogeneity and differences in community size could be another potential explanation for the observed high beta diversity (58). However, hydrologic data paired with the fact that the null ecological modeling method accounts for differences in alpha diversity (60) indicate that model results are accurate and that the high observed beta diversity is the result of ecological drift.

The influence of selection is also clear throughout the site. The metacommunity (average sitewide NTI = 1.73), as well as local communities sampled through time, show significant phylogenetic clustering, indicating that microorganisms within the aquifer are highly adapted to environmental conditions (see Fig. S7 in the supplemental material). While it is unlikely that this observation is solely due to high pH adaptations, pH is known to be a significant driver of microbial community composition (33, 61), and high pH conditions and the physiological adaptations required to survive under these conditions likely played a significant role in structuring the observed microbial communities at CROMO. Microbial community diversity measures are also significantly affected by pH conditions (Fig. 5), and metagenomic and metatranscriptomic data indicate that pH homeostasis mechanisms used in alkaline conditions are prevalent and actively transcribed within the wells (Fig. 6; see Table S7 at https://doi.org/10.6084/m9.figshare.14372030). The increased abundance and transcription of genes associated with motility at high pH, including flagellar synthesis genes, and type IV pili (Fig. 6B, D and E, G and H, and L) potentially represent a means to improve access to both limited available DIC and oxidants in the deep subsurface or to enable the attachment of organisms to minerals to form biofilms and/or aid in electron transport to mineral surfaces. Improved access to substrates via motility or adhesion and the capability to form biofilms that can lower pH within the microenvironment (62) may be key survival strategies that allow microbial populations to persist under hyperalkaline conditions in the deep subsurface. Additionally, factors such as DO concentrations (Table 2), redox conditions (Fig. S6A to F), salinity (Fig. S5A to I), temperature (Fig. S6M to V), and DIC concentrations (Table 2; Fig. S6G to L), which are likely controlled by pH, and other unmeasured variables likely play a smaller role in driving community composition differences across the wells (Table 2; see Table S6 at https://doi.org/10.6084/m9.figshare.14983869). While ecological modeling results did not identify homogeneous selection as the dominant assembly process across the site (see Table S2 at https://doi.org/10.6084/m9.figshare.14983857), other measures of microbial community diversity (Fig. 5; see Table S2 at https://doi.org/10.6084/m9.figshare.14983857; see Table S6 at https://doi.org/10.6084/m9.figshare.14983869), phylogeny (Fig. S4 and S7), and physiology (Fig. 6; see Table S7 at https://doi.org/10.6084/m9.figshare.14372030) indicate that pH plays a major role in structuring microbial community composition in the CROMO aquifer.

Given the strong evidence for selection and dispersal limitation within the physicochemical and microbial community data, the preponderance of undominated processes at the site was initially surprising. However, recent studies of soil (33) and a fractured shale aquifer (34) using the Stegen et al. (28) ecological modeling framework provide some insight. Tripathi and colleagues (33) showed that while pH plays a significant role in structuring soil microbial communities, selection imposed by pH accounted for only ∼17% of observed assembly. As in our study, this low value indicates that other assembly processes still play a substantial role in structuring microbial communities under extreme pH conditions. Recent work by Danczak and colleagues (34) in a fractured shale aquifer highlighted how strong concurrent homogeneous selection and variable selection counteract to result in an undominated community assembly signal. While this same mechanism is not observed at CROMO, results from Danczak and colleagues (34) demonstrate that an undominated assembly signal can be the result of strong counteracting processes. Extreme pH and slow fluid flow at CROMO consistently impose strong counteracting deterministic and stochastic processes, resulting in an undominated assembly signal observed across the site (Fig. 3E).

Our observations at CROMO are also supported by recent computational and experimental work that has shown that dispersal conditions and microbial community size affect the importance of selection in structuring microbial communities (30, 59). Strong environmental selection can result in low diversity and low biomass communities that are inherently prone to enhanced drift as theorized by Vellend (23) and observed in computational modeling experiments by Evans and colleagues (30). Ecological drift in communities assembled under strong selection can be further enhanced under low dispersal conditions (30, 59), resulting in microbial communities composed of a small number of highly abundant species, with large variation in the birth and death rates of rare taxa within the communities (59). Microbial communities characterized in serpentinizing systems thus far have notoriously low diversity and are primarily composed of a few dominant taxa and many highly variable low abundance species (6, 15, 16, 22, 44), which likely makes serpentinite microbial communities inherently prone to enhanced ecological drift.

Finally, we propose a more nuanced mechanism of assembly within the serpentinizing subsurface, relevant to the geological history (63) and long groundwater residence times in the region (64). Poor connectivity to the surface (see Table S4 at https://doi.org/10.6084/m9.figshare.14983863) and slow fluid flow in the subsurface (Fig. 4; see Table S5 at https://doi.org/10.6084/m9.figshare.14983866) indicate that dispersal of microorganisms into and out of the region is extremely slow. Microorganisms present within the serpentinizing subsurface were therefore potentially transported to the terrestrial surface when the ophiolite was obducted and continued to grow and persist (22, 65) or were introduced to the system by persisting hundreds or thousands of years along slow regional groundwater flow pathways, similar to observations of community assembly patterns in deep seafloor sediments (52). In either case, extreme pH conditions buffered by the presence of serpentine minerals present throughout much of the ophiolite (66) impose strong environmental filtering and likely select for well-adapted alkaliphilic microorganisms long before they reach the subsurface localities observed in this study (52). Within this geologic context, we suggest that homogeneous selection imposed by extreme pH structures a low diversity alkaliphilic metacommunity within the larger region of the CROMO aquifer from which the observed local communities in this study are assembled. Within observed local communities, homogeneous selection maintains the persistence of dominant alkaliphilic microbial community members. At the same time, poor dispersal between local communities and variability in the birth and death rates of the large pool of rare community members results in enhanced ecological drift over space and time (23, 30, 59). Overall, these results highlight the important roles and complex interplay that occurs between selection, dispersal, and ecological drift in structuring subsurface microbial communities. Given the great heterogeneity observed in subterranean environments, it is critical to continue to assess community assembly processes in these systems to better constrain how different physical and environmental conditions structure microbial communities within the expansive subsurface biosphere.

## MATERIALS AND METHODS

### Site description and sample collection.

CROMO is located within the McLaughlin Natural Reserve near Lower Lake, CA. This site lies within the Coast Range Ophiolite and consists of four wells drilled >30 years ago and eight wells drilled in August of 2011. The latter set of wells was drilled using techniques designed to minimize and quantify subsurface contamination (40). Initial characterization of CROMO fluids indicated a strong influence of serpentinization (low Eh, pH > 11) and microbial communities that share similarity to other characterized serpentinite springs (11, 15, 40). The main aquifer is confined by a well-cemented aquitard (41), and tritium analyses, described below, indicate no mixing of modern surface water within the aquifer (53, 67, 86). CROMO has been sampled two or three times per year since the site was established in 2011, resulting in a high-resolution time series data set of geochemistry and microbial biodiversity. The sampling procedure utilized to collect fluids from CROMO wells has been previously described (11, 15). Geochemical and microbial sample collection methods are described in detail in Text S1 in the supplemental material.

### Topographic profile and cross section construction.

A topographic profile and cross section of the study area (Fig. 4) were created using ArcMap (Esri, Redlands, CA, USA), and Adobe Illustrator (Adobe, San Jose, CA, USA). Well head locations and elevations were obtained as described in Text S1 and drawn into the cross section at their proper location and depth. Well head coordinate and elevation data are listed in Table S10 (https://doi.org/10.6084/m9.figshare.14983893). A detailed description of topographic profile methods and assumptions made in the construction of the cross section can be found in Text S1. Detailed maps displaying the location of McLaughlin Reserve in California, a geologic map of the CROMO site, and the location of each well cluster at the site can be found in the article by Ortiz et al. (41).

### Estimation of aquifer properties.

Aquifer properties were estimated using the program AQTESOLV (68). Drawdown/Recovery analysis was performed within AQTESOLV to obtain estimates of transmissivity (*T*), and storativity (*S*) to calculate hydraulic conductivity (*K*) using *T* and aquifer thickness (*b*) according to the following equation: *T* = *K* × *b* (see Table S5 at https://doi.org/10.6084/m9.figshare.14983866). Detailed description of data collection, model parameters (83), and fitting model solutions (84, 85) can be found in Text S1.

### Tritium sample collection.

Water samples for tritium analyses were collected in June 2017. Water was collected into 0.5-liter narrow-mouth high-density polyethylene bottles. Samples were air-tight, and bottle mouths were wrapped with parafilm to prevent evaporation or leakage from the bottles. Samples were stored at room temperature and shipped to University of Waterloo Environmental Isotope Laboratory (UW-EIL, Waterloo, ON, Canada). Detailed methods of tritium analyses performed by UW-EIL are described in Text S1. Measured tritium values for each well are included in Table S4 (https://doi.org/10.6084/m9.figshare.14983863).

### Geochemical analyses.

Analytical methods used to measure DIC have been previously described (11, 15). Briefly, fluid samples were acidified to convert all forms of DIC to carbon dioxide (CO_2_), which could partition into the headspace of the sealed sample vial. Subsequently, the concentration of CO_2_ in the headspace gas was quantified by gas chromatography with flame ionization detection (FID) (SRI 8610C; SRI Instruments, Torrance, CA, USA), using an inline methanizer to convert liberated CO_2_ to methane prior to passage through the FID. All analyses were performed in duplicate. Geochemical data used for analyses can be found in Table S1 (https://doi.org/10.6084/m9.figshare.14983851).

### Extraction of DNA and RNA.

Extractions of DNA and RNA from 0.22-μm Sterivex filter cartridges were performed as previously described by Twing and colleagues (15) and Sabuda and colleagues (22), respectively. A brief description can be found in Text S1.

### Sample preparation, sequencing, and data analysis of metagenomes and metatranscriptomes.

Metagenomic and metatranscriptomic sequences were previously reported (15, 20, 22, 88). Sample preparation and sequencing of metagenomes and metatranscriptomes were carried out as previously described (22). The assemblies and predicted protein annotations reported here were performed as previously described by Sabuda and colleagues (22).

### 16S rRNA gene amplicon sequencing.

Throughout the course of the project, samples were submitted for amplicon sequencing of the V4 region of the 16S rRNA gene at three different sequencing centers: Department of Energy Joint Genome Institute (JGI), Marine Biological Laboratory’s (MBL) Josephine Bay Paul Center, and the Michigan State University (MSU) Genomics Core Facility (see Table S11 at https://doi.org/10.6084/m9.figshare.14983896). Sequences generated by the JGI were reported by Twing and colleagues (15). Sequences generated by the MBL were reported by Crespo-Medina and colleagues (11). Samples sequenced at the MSU Genomics Core were submitted for sequencing of the V4 region of the 16S rRNA gene. Amplification, quantification, and sequencing procedures performed by the MSU Genomics Core have been previously described (22). Blanks and extraction blanks collected alongside samples in this data set could not be quantified or amplified and were not submitted for sequencing.

### 16S rRNA sequence processing.

Sequences generated by JGI, MBL, and the MSU Genomics Core were processed using mothur v1.39.5 (69) as previously described (11, 15, 22). Quality-trimmed fasta files and count tables for sequences from each sequencing center were concatenated together and clustered into OTUs at a 3% distance threshold using the *de novo* distance-based greedy clustering (DGC) method as implemented in mothur v1.39.5 (70). *De novo* clustering has successfully been used to compile and reanalyze 16S rRNA data from multiple sources for large meta-analysis studies of the human microbiome (71, 72). Continued discussion on the successful use of DGC to cluster sequences from different sequencing centers and analyses of sequences clustered using this method are included in Text S1. Clustered OTUs were aligned to the SILVA SSURef alignment (v132), and taxonomic classifications were assigned using mothur.

Following the successful merger of the 16S rRNA data sets (23,994 OTUs and 6,210,850 reads), count data from sample replicates were averaged and rounded to the nearest whole count number to avoid statistical issues that can arise from pseudoreplication (73, 74). Singletons, which rounded to zero when present within a set of replicates, were removed (8,526 OTUs), and sequences identified as eukaryotes (58 OTUs and 1,475 reads), archaea (138 OTUs and 7,271 reads), mitochondria (46 OTUs and 1,295 reads), chloroplasts (94 OTUs and 7,881 reads), and unknown (842 OTUs and 9,127 reads) by SILVA were removed. Following this process, the 16S rRNA sequences were screened for potential contaminants associated with DNA extraction reagents and for human skin and feces-derived organisms previously identified in deep subsurface samples by Sheik and colleagues (75). Following analysis of the taxonomy and distribution of each potential contaminant within the data set, sequences that were likely contaminants were removed from the data set (792 OTUs and 111,576 reads). In addition to this cleanup, two human contaminant microorganisms, Simkania negevensis (76) and Akkermansia muciniphila (77), were removed that were identified at high abundance in a handful of samples. These organisms from families cvE6 and *Akkermansiaceae* accounted for a maximum of 39% and 48% of the reads in the affected samples, respectively, and 1.6% of total reads from the data set. There were no patterns in sample contamination by these two organisms when looking at sample collection or sample extraction logs. A similar approach to removing contaminant sequences from a deep subsurface data set was successfully used by Fullerton and colleagues (78) and Sabuda and colleauges (88). The final 16S rRNA data set used for analysis consisted of 13,444 OTUs and retained 96% of the original reads (5,974,056 reads). Following data set filtering, a phylogenetic tree was generated using FastTree v2.1.3 (79).

Excel files containing raw count table data, averaged and contaminant filtered data, and count and taxonomy information for removed OTUs (https://doi.org/10.6084/m9.figshare.14879535 and https://doi.org/10.6084/m9.figshare.14371964) are available on Figshare along with R code used to identify and screen potential contaminant OTUs (https://figshare.com/projects/Community_Assembly_in_Serpentinizing_Ophiolites/101648).

### Statistical analyses.

Basic statistical analyses and data exploration of the community data and associated metadata were performed using the R packages phyloseq and vegan (80, 81). These packages were used to combine and organize 16S rRNA tag sequencing data with corresponding sample metadata and to perform basic data visualization and data analysis. Correlations between the ecological modeling matrices and environmental variables were performed using Mantel tests, using the command mantel.rtest() from the R package ade4 (82). PERMANOVA analyses were performed in vegan using the adonis() function (28). PERMANOVA results were used to define significant relationships between community data and environmental variables. Sample richness was calculated using the function specnumber() from the R package vegan (81). Pielou’s evenness was calculated by dividing the Shannon diversity index, calculated using the function diversity() in vegan (81), by sample richness, which was previously calculated. Geochemical data, richness and Pielou’s evenness data, and metagenomic and metatranscriptomic data were plotted in ggplot(), and linear regression models were calculated using the lm() function.

### Ecological modeling.

Ecological modeling of community and phylogenetic turnover within the system was performed according to the framework developed by Stegen and colleagues (28). A basic assumption of the framework is that there is significant phylogenetic signal (i.e., closely related organisms have similar habitat preferences) at short phylogenetic distances, making the use of the βNTI metric, which assesses phylogenetic turnover between close relatives, an ideal measure for the framework (60). Mantel correlograms were used to test for phylogenetic signal at different phylogenetic distance classes. Positive, significant correlations at short phylogenetic distances were observed when pH environmental niches were assessed (see Fig. S4 in the supplemental material), indicating that the community assembly framework developed by Stegen and colleagues (28) was an appropriate choice for the data set analyzed here.

The framework developed by Stegen and colleagues uses two different metrics to quantify the contributions of selection, dispersal, and ecological drift that contribute toward an observed assembled community (28). This model can quantify all processes except for speciation/diversification as has been outlined in Vellend’s seminal paper that conceptualizes the processes of community assembly (23). The Stegen et al. framework (28) first assesses the role of selection by assessing changes in phylogenetic distance using beta-mean nearest taxon distance and βNTI for all pairwise comparisons within the data set. Once the roles of selective processes have been assessed, pairwise comparisons that were not significant with the βNTI metric are assessed with the RCbray metric to look at the role of the stochastic processes of dispersal and drift (28). Detailed description of the model and how each metric is calculated are included within Text S1.

All R scripts, excel, and csv files used in analyses are available on Figshare at https://figshare.com/projects/Community_Assembly_in_Serpentinizing_Ophiolites/101648.

### Data availability.

The 16S rRNA gene sequence data used in this work are publicly available in the NCBI Sequence Read Archive (SRA) under the BioProject accession number PRJNA690585. CROMO metagenome sequences previously published by Twing and colleagues (15) are publicly available in the JGI IMG/M database under the project identifiers (IDs) 1021918, 1021921, 1021924, and 1021927 and in the MG-RAST database under the following sample IDs: 4569549.3, 4569550.3, 4569551.3, and 4569552.3. Metagenome sequences previously published by Seyler and colleagues (20) are publicly available under the BioProject IDs: PRJNA410019, PRJNA410020, PRJNA410022, PRJNA410035, PRJNA410037, PRJNA410553, PRJNA410555, PRJNA410036,PRJNA410024, PRJNA410028, PRJNA410025, PRJNA410023, PRJNA410027, and PRJNA410026. Metagenomic sequences previously published by Sabuda and colleauges (88) are publicly available in the NCBI SRA database under the following accession IDs: SRX9385611, SRX9385612, and SRX9385613. CROMO metatranscriptome sequences previously published by Sabuda and colleagues (22) are publicly available in the SRA under the following accession IDs: SRX3339504, SRX3339503, SRX3339089, SRX3331179, SRX3331177, SRX3330963, SRX3330943, and SRX3330753.

10.1128/mSystems.00300-21.9 FIG S8 Multidimensional scaling plot of CROMO 16S rRNA microbial community samples using Morisita-Horn distances. Points represent individual samples and are colored by the sequencing center they were sequenced at. Points that lie closer together are more similar to each other; points further apart are more dissimilar. The overlap and consistent intermixing of samples from different sequencing centers indicate that samples are not biased based on the sequencing center they were sequenced at and are representative of the samples themselves. Download FIG S8, EPS file, 1.5 MB. Copyright © 2021 Putman et al. 2021 Putman et al. https://creativecommons.org/licenses/by/4.0/ This content is distributed under the terms of the Creative Commons Attribution 4.0 International license.

10.1128/mSystems.00300-21.10 FIG S9 Heatmap displaying Bray-Curtis similarity data between samples where technical replicates were sequenced at more than one sequencing center. Dark red colors indicate high similarity, while light red colors indicate low similarity in community composition. Comparisons between technical replicates from the same sampling trip and same well can be seen along the diagonal of the heatmap. Dark red values, indicating high community composition similarity, are observed along the diagonal, indicating that community composition and taxonomic structure of technical replicates from the same well were not significantly altered based on the sequencing center the sample was sequenced at. Download FIG S9, EPS file, 1.5 MB. Copyright © 2021 Putman et al. 2021 Putman et al. https://creativecommons.org/licenses/by/4.0/ This content is distributed under the terms of the Creative Commons Attribution 4.0 International license.
