# Supplementary material for: Microbial Communities in a Serpentinizing Aquifer Are Assembled through Strong Concurrent Dispersal Limitation and Selection
Source: mSystems. 2021 Sep 14;6(5):e00300-21. doi: 10.1128/mSystems.00300-21 (PMC8547479; doi:10.1128/mSystems.00300-21)
Supplement: TEXT S1 [file msystems.00300-21-s0001.docx]

**­­Supplementary Information for**

Microbial communities in a serpentinizing aquifer are assembled through strong concurrent dispersal limitation and selection.

Lindsay I. Putman, Mary C. Sabuda, William J. Brazelton, Michael D. Kubo, Tori M. Hoehler, Tom M. McCollom, Dawn Cardace, and Matthew O. Schrenk

Matthew O. Schrenk

Email: schrenkm@msu.edu

**S.I. Appendix**

**S.I. MATERIALS & METHODS:**

*Sample collection:*

Fluids were pumped to the surface using permanently emplaced positive displacement Teflon bladder pumps (Geotech Environmental Equipment, Denver, CO, USA), and flushed through a YSI 3059 flow-through cell attached to a YSI multiprobe (Yellowsprings, OH, USA). The flow-through cell measured pH, oxidation-reduction potential (ORP), dissolved oxygen (DO), specific conductance, and temperature (11,15). Fluid sampling from tubing attached directly to the flow-through cell began following the stabilization of fluid temperature and DO. Fluid samples to measure dissolved inorganic carbon (DIC) were collected by attaching syringes directly to the tubing attached to the flow-through cell, allowing for anoxic sampling of the fluids. DIC samples were filtered through a 0.22-μm syringe filter into an acid washed serum vial filled with N_2_ and a pre-set volume of phosphoric acid (11,15). Fluids for molecular microbiology samples were collected in sterile 4-liter cubitainers, filtered through 0.22-μm Sterivex filters (Merck Millipore, Billerica, MA, USA) using a Masterflex E/S portable sampler (Masterflex, Gelsenkirchen, Germany) and compatible sterile tubing and adaptors, and flash frozen in liquid nitrogen in the field. Upon return to the lab flash frozen Sterivex filters were stored at -80°C (15).

*Well head GPS Measurements:*

         Elevation and GPS coordinates were collected in January of 2016 for all twelve CROMO wells to obtain accurate measurements for spatial analyses. A Trimble GNSS system (California Surveying and Drafting Supply (CSDS), Dublin, CA) was attached to a tripod balanced precisely over the center of each well head for 20 minutes. Elevation and GPS coordinates were also collected for nearby creek beds at each well cluster. Data obtained by the unit was post-processed by CSDS using CORS site P206 CRAZYCREEK and US State Plane 1983 Datum California Zone 2 using Geoid 12B. Elevation measurements were adjusted accordingly to account for well monument height above the ground and any additional space between the monument head and the tripod. Collected elevation and location data were used in the development of a topographic profile of the aquifer and surrounding landscape, and in the estimation of aquifer properties. Elevation and coordinate data are listed in **Table S10** (https://doi.org/10.6084/m9.figshare.14983893).

*Topographic Profile and Cross Section Construction:*

Well head locations and elevations were obtained as described above. These data were used to create a shape file of well locations within ArcMap (Esri, Redlands, CA, USA). Following this, an ArcMap compatible version of the Jericho Valley Quadrangle California 7.5 Minute Series (Topographic) map was obtained from the U.S. Geological Survey (USGS) Data Catalog (USGS, Reston, VA, USA). A digital elevation model (DEM) was available for this location, but at extremely low resolution. Due to this, the historic USGS quadrangle map was used instead. The CROMO well clusters are located within a V-shaped valley and lie approximately 1.4 km away from each other. A 2.3 km cross section line was drawn from west to east between start and end point features in ArcMap across the valley, intersecting the well cluster locations. Point features were added along the line each time it intersected a topographic line, and well locations were added to the line as well. The wells did not all fall directly on the topographic line, so their location was approximated on the line based on their order from west to east. The map data were projected using the North American 1983 geographic coordinate system and datum. The map was then exported from ArcMap as an Adobe Illustrator (Adobe, San Jose, CA, USA) file.

         In Adobe Illustrator, the base map was deleted, and the cross section line and intersecting points were grouped together as a single object. This object was then rotated to lie horizontally to create a topographic profile. The grid function was used to generate a uniform grid on the file to create an accurate profile. A vertical exaggeration of 10x was chosen for the vertical axis to display the topographic profile as well as geologic layers and well depths most accurately in the subsurface. Topographic profile points were drawn at the proper elevation on the profile based on their horizontal location on the original cross section line. Lines were drawn between the points to mimic the natural shape of the landscape.

         Following the creation of the profile, subsurface geological layers were added to the cross section. Ortiz and colleagues (41) characterized the relevant water-bearing units within the subsurface at both CROMO well cluster sites using electrical resistivity tomography (ERT). The subsurface ERT maps indicate a shallow topsoil water-bearing unit, followed by an aquitard, and then a main serpentine aquifer (41). While the thickness of the topsoil layer varied by a few meters between the two well cluster locations, the thickness of the aquitard and serpentine unit below appear to be relatively uniform in thickness at both locations (41). Given the uniform presence of all three layers at both locations, it was assumed that the upper soil layer, aquitard, and serpentine unit were laterally continuous and of uniform thickness throughout the extent of the cross section. The main aquifer was assumed to be 76.2 m in thickness, accommodating the deepest well at the site. The depth at which basement rock lies in the area is unknown, but it is assumed that basement rock likely serves as an aquitard at the bottom of the main serpentine aquifer unit (41). Lines were drawn to accurate lengths to represent individual wells. The lines were then emplaced in the proper location on the topographic profile, providing accurate representation of the well’s relationships to each other in the subsurface in the context of the natural topography of the landscape. Fluid flow direction lines were placed in the main aquifer unit moving from west to east. It is assumed that groundwater generally moves from topographic high to topographic low and moves down elevation within the valley from the QV well cluster to the CSW well cluster. Hydraulic conductivity estimates (K) (see methods below) are included at each well cluster.

*Estimation of Aquifer Properties:*

Displacement data during pumping and recovery was obtained from measurements collected by ODYSSEY temperature and pressure transducers emplaced within the wells (Dataflow Systems Ltd., Christchurch, New Zealand). Water elevation data obtained by the transducers was pulled from the timeframe during pumping and for 24 hours following pumping. Water elevation data was converted to displacement data, compatible for analyses in AQTESOLV (68), by subtracting elevation data from the starting depth to water elevation level, prior to when the system was disturbed by pumping activity. The time in minutes from the start of pumping was also calculated for each displacement measurement. The time and displacement data were then imported into AQTESOLV.

Model parameters are as follows: aquifer thickness was assumed to be 76.2 m. The hydraulic conductivity anisotropy ratio was set at 0.1 based on average estimates obtained by Marechal and colleagues (83) on a variety of hard rock aquifers. The radii of the positive displacement Teflon bladder pumps (Geotech Environmental Equipment, Denver, CO, USA) are 2.01 cm, based on the reported diameter of the pumps from the manufacturer. The inner radii of fully cased wells (CSW1.2, CSW1.3, CSW1.4, CSW1.5, QV1.2, and QV1.3) are 2.53 cm, and the radii of entire wells, including PVC piping is 3.13 cm, based on the thickness of Sch40 PVC piping used to case the wells. The inner radii of the partially cased wells are 6.34 cm and 5.06 cm for CSW1.1 and QV1.1 respectively, and the radii of the whole wells are 6.94 cm and 5.67 cm for CSW1.1 and QV1.1. Since the main aquifer being sampled lies beneath an aquitard, wells were characterized as vertical partial penetration wells. Wells were then characterized based on their depth from the top of the aquifer layer to the top of the screened interval. The top of the aquifer was set to 13 m in the subsurface based on thickness estimates of the topsoil layer and aquitard from Ortiz and colleagues (41). In cased wells, the screened interval is 1.5 m, except for CSW1.3 which has a screened interval of 2.6 m. In the partially cased wells, the uncased portion was assumed to be the screened interval and is 4.27 m and 10 m from the top of the aquifer in CSW1.1 and QV1.1, respectively. Pumping rates (L/min) were calculated using the length of pumping cycles used at each well for a given sampling trip, the total time pumping at each well, and total volume of water removed while pumping. The total volume of water removed was divided by the number of pump cycles completed during the total sampling time to obtain a pump rate (L/min). This pump rate was set for the duration of the timeframe sampling occurred and served as the drawdown portion of the test. The pumping rate for recovery data is set to 0 L/min.

Following the input of well and aquifer parameter data and time-displacement data, a time-displacement graph was generated to fit a model solution. The Theis(1935)/Hantush(1961) solution (84,85) for non-leaky confined aquifers was used to fit time-displacement curves for all the wells. While this aquifer does contain fractured rock, a solution for fractured rock aquifers was not used, as the type and extent of fracturing has not been characterized at CROMO. The aquifer also contains interbedded layers of clay material (40), so the simplest solution for a confined aquifer was used as the model solution. Curve fit solutions provide estimates for transmissivity (T) and storativity (S). T and aquifer thickness (b) were then used to calculate hydraulic conductivity (K) using the equation T = K × b (**Table S5** at https://doi.org/10.6084/m9.figshare.14983866).

*Tritium Analysis:*

The University of Waterloo Environmental Isotope Laboratory (UW-EIL) performed enriched tritium analyses on water samples from each well in June of 2017.  Water samples were enriched fifteen times via electrolysis and then tritium was quantified using the liquid scintillation counting technique.  The detection limit with these methods is 0.8 ± 0.8 T.U. at low levels, where 1 T.U. is equivalent to one tritiated water molecule per 10^18^ water molecules (86).  UW-EIL runs three background samples with each batch of samples: a natural groundwater with no detectable tritium, lab deionized water, and a standard traceable to NIST-4926-E. Measured tritium values for each well are listed in **Table S4** (https://doi.org/10.6084/m9.figshare.14983863).

*DNA and RNA Extraction:*

Cells on filter cartridges for DNA extraction were lysed by using freeze/thaw cycles and lysozyme/Proteinase K treatment and purified using a series of phenol/chloroform extractions. DNA was precipitated using ethanol and purified using the Genomic DNA Clean & Concentrate kit (Zymo Research, Irvine, CA, USA) (15). Genomic DNA was quantified using the High Sensitivity dsDNA Assay Kit on a Qubit fluorometer (Invitrogen, Carlsbad, CA, USA). RNA extractions were performed as previously described (22), using a modified phenol/chloroform extraction method. RNA was precipitated using ethanol, suspended in RNAse-free water, and quantified using a Qubit fluorometer (Invitrogen, Carlsbad, CA, USA) (22).

*Ecological Modeling Framework:*

The ecological modeling framework developed by Stegen and colleagues (28) was used to quantify the role that selection, dispersal, and drift processes play at CROMO. Null modeling results using the β-nearest taxon index (βNTI) and the modified Raup-Crick (RCbray) metric, described below, result in distance matrix-like output where pairwise βNTI and RCbray values have been calculated between all samples included within the data set. This resulted in square distance matrix-like objects that consisted of 104 rows and 104 columns for this data set. As is seen in the calculation of other distance matrices, the upper and lower triangles of the distance matrix are identical and the diagonal is populated by values of 0 or 1 indicating that samples are identical to one another when a sample is compared to itself. The βNTI and RCbray distance matrices obtained from ecological modeling are available for download on Figshare (https://figshare.com/projects/Community_Assembly_in_Serpentinizing_Ophiolites/101648).

Selection processes are distinguished from stochastic (random) processes first in the model and are quantified using the β-mean nearest taxon distance (βMNTD) and βNTI metrics. First, βMNTD is calculated for all pairwise comparisons within the data set to assess phylogenetic contributions to differences in community composition across the data set. Following this, a null modeling approach permutes taxa locations on the phylogenetic tree 999 times, generating a distribution of βMNTD values under random assembly (i.e., under completely random assembly we would assume that community members are randomly related to each other). Following this, βNTI is calculated by comparing observed βMNTD values to the null βMNTD distribution and determining the deviation of the observed βMNTD from the null expectation. βNTI results are compiled for each pairwise comparison and are used to assess phylogenetic turnover between wells and over time. Values of |βNTI| > 2 indicate that deterministic (selective) processes are responsible for differences in community composition between two locations/timepoints, while |βNTI| < 2 indicates that stochastic processes are responsible for differences between the two samples (37). Values of |βNTI| > 2 can be further analyzed by assessing the raw results, which can range from βNTI  ≥ 2 or βNTI ≤ -2. βNTI  ≥ 2 indicates variable selection, where different environmental conditions between two locations select for different microbial community compositions. βNTI ≤ -2 indicates homogeneous selection, where environmental conditions drive communities to more homogeneous compositions (28). Both environmental conditions and biotic interactions can impose selection on microbial communities (24). Pairwise comparisons of |βNTI| < 2, which implicate the role of stochastic processes, are further analyzed using an abundance weighted Raup-Crick measure to quantify the roles of the stochastic processes of dispersal and ecological drift (28).

Stochastic processes are quantified using the RCbray metric, which incorporates species abundance data to the metric originally developed by Chase and colleagues (26,60). The stochastic or random processes of microbial community assembly that are quantified here are dispersal and ecological drift (24). Dispersal here is considered passive and depends upon how environmental conditions allow for the transport or lack of transport of microorganisms through a natural environment. Ecological drift is the result of random changes in the death and replication rate of microorganisms, which ultimately leads to variance in the presence and abundance of different microorganisms (56). A null modeling approach permutes community composition 999 times and calculates a Bray-Curtis dissimilarity for each pairwise comparison. This generates a  null distribution of Bray-Curtis values based on operational taxonomic unit (OTU) richness and diversity within each sample. Observed Bray-Curtis values are compared to the null distribution to determine how the observed value deviates from the null expectation. Results are normalized for all pairwise comparisons to generate an RCbray metric that ranges from +1 to -1 (60). Values of |RCbray| >0.95 for any given pairwise comparison indicates that either homogenizing dispersal or dispersal limitation paired with ecological drift drives differences in observed community composition. Dispersal limitation (RCbray > 0.95) occurs when turnover between communities is greater than would be expected when considering ecological drift alone. This indicates that lack of interaction between communities due to dispersal limitation paired with ecological drift is driving differences in observed community compositions (60).  Homogenizing dispersal (RCbray < -0.95) occurs when turnover between communities is less than would normally be expected. This can occur when dispersal of microbial community members is high and local selection in an area is low. Continual transport of microorganisms between two locations ultimately generates community compositions that are more similar to each other than would be expected by chance (56,60). Only pairwise comparisons that were not significant with the βNTI metric (|βNTI| < 2) were analyzed with the RCbray metric. This leaves out pairwise comparisons dominated by selection (|βNTI| > 2) and uses the RCbray metric to quantify the stochastic process in pairwise comparisons that are not driven by selection processes (|βNTI| < 2).

Model results where both βNTI and RCbray are nonsignificant (|βNTI| < 2 and |RCbray| < 0.95) are defined as undominated, where values for βNTI and RCbray do not cross the significance threshold. In this scenario, both deterministic and stochastic processes are at work, but neither can be deemed entirely responsible for observed differences in pairwise comparisons of communities. Undominated assembly has been posited to occur under weak selection and moderate dispersal (28,56) and has also been observed to be the result of strong interactions between variable and homogeneous selective processes (34). Importantly, undominated assembly does not indicate that neither stochastic or deterministic processes are at play, instead it reveals that no single ecological process can explain the observed variation between communities and that assembly occurs through a mixture of stochastic and deterministic processes that cannot be well defined by the model. Ecological modeling results are presented in **Table S2** (https://doi.org/10.6084/m9.figshare.14983857).

**S.I. DISCUSSION:**

*Homogenizing Dispersal in Partially Uncased Wells:*

Wells CSW1.1 (4.26 m of 19.5 m uncased) and QV1.1 (21.2 m of 23 m uncased) are partially cased well. Wells are generally cased with an impermeable material (PVC piping) to help support the sides of the borehole and to allow for water to be pumped from a discrete depth range at the bottom of the well, called the screened interval. The screened interval (commonly 1-3 m) allows fluid to enter the borehole and be pumped out of the well. Due to their uncased nature, hydraulic gradients generated during pumping activity in CSW1.1 and QV1.1 likely pull and mix fluids from the top of the uncased portion down to the bottom of the well where fluids are being pumped (87). Samples collected from these wells likely represent a greater depth range in the formation as compared to fluids collected from the more discrete 1.5 m screened intervals present on cased wells at the site. As a result, sampled communities from the partially uncased wells likely represent a mixed microbial community, representative of a much larger depth range. This results in a homogenizing dispersal signal (RCbray < -0.95) between the uncased wells and cased wells at nearby depths (1-10 m shallower or deeper).

This phenomenon likely alters the interpretation of results seen in **Figure 3D**. CSW1.1 and QV1.1 are both included within the extreme pH well grouping and are consistently characterized by extremely high pH (**Fig. 1B**). Enhanced homogenizing dispersal observed in these wells that is the result of top-down fluid dynamics in the uncased portion of these wells (87) results in an increased contribution of homogenizing dispersal in extreme pH wells that is not in line with hydrological data obtained from the site (**Table S5** at https://doi.org/10.6084/m9.figshare.14983866).

*Use of Distance-based Greedy Clustering in 16S rRNA Sequence Processing:*

Distance-based Greedy Clustering (DGC) was chosen because it uses the distance between sequences within a data set to cluster them into OTUs, as opposed to comparing the distance of sequences to that of sequences in a reference database, as is done when using closed-reference clustering methods (70). The DGC clustering method prevented sequences from different sequencing centers from clustering separately from each other. *De novo* clustering methods have been successfully used to cluster OTUs from data compiled from different sequencing platforms (71,72). Samples sequenced at the different sequencing centers cluster together by each different well, as has been seen with smaller subsets of the data set generated at a single sequencing center in the past (15,22). Evidence of this can be seen in **Figure S8**. This is further supported by the fact that several samples were sequenced at two of the three sequencing centers (**Fig. S9; Table S11** at https://doi.org/10.6084/m9.figshare.14983896) used in this study. These samples overlap with each other by sample (**Fig. S8**), show high community composition similarity (**Fig. S9**), as would be expected for the same sample, and do not differ from each other based on the sequencing center (**Figs. S8 & S9**).

**S.I. References**

In text-citations in the S.I. text are numbered based on the reference list found in the main manuscript. Please refer to the works cited in the main manuscript.
