# Supplementary figures and images for: Microbial Communities in a Serpentinizing Aquifer Are Assembled through Strong Concurrent Dispersal Limitation and Selection
Source: mSystems. 2021 Sep 14;6(5):e00300-21. doi: 10.1128/mSystems.00300-21 (PMC8547479; doi:10.1128/mSystems.00300-21)

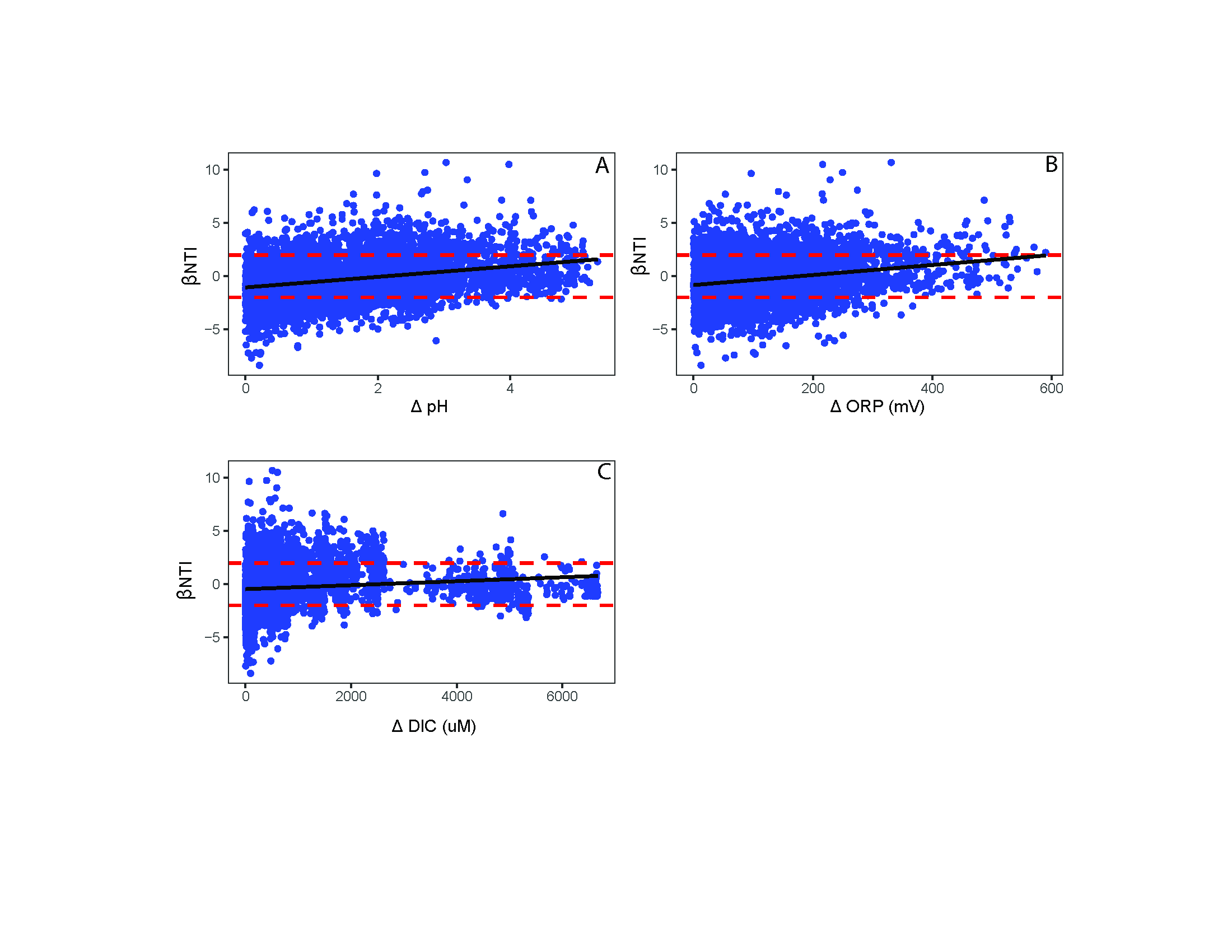

Supplement: FIG S2 [file msystems.00300-21-sf002.tif]

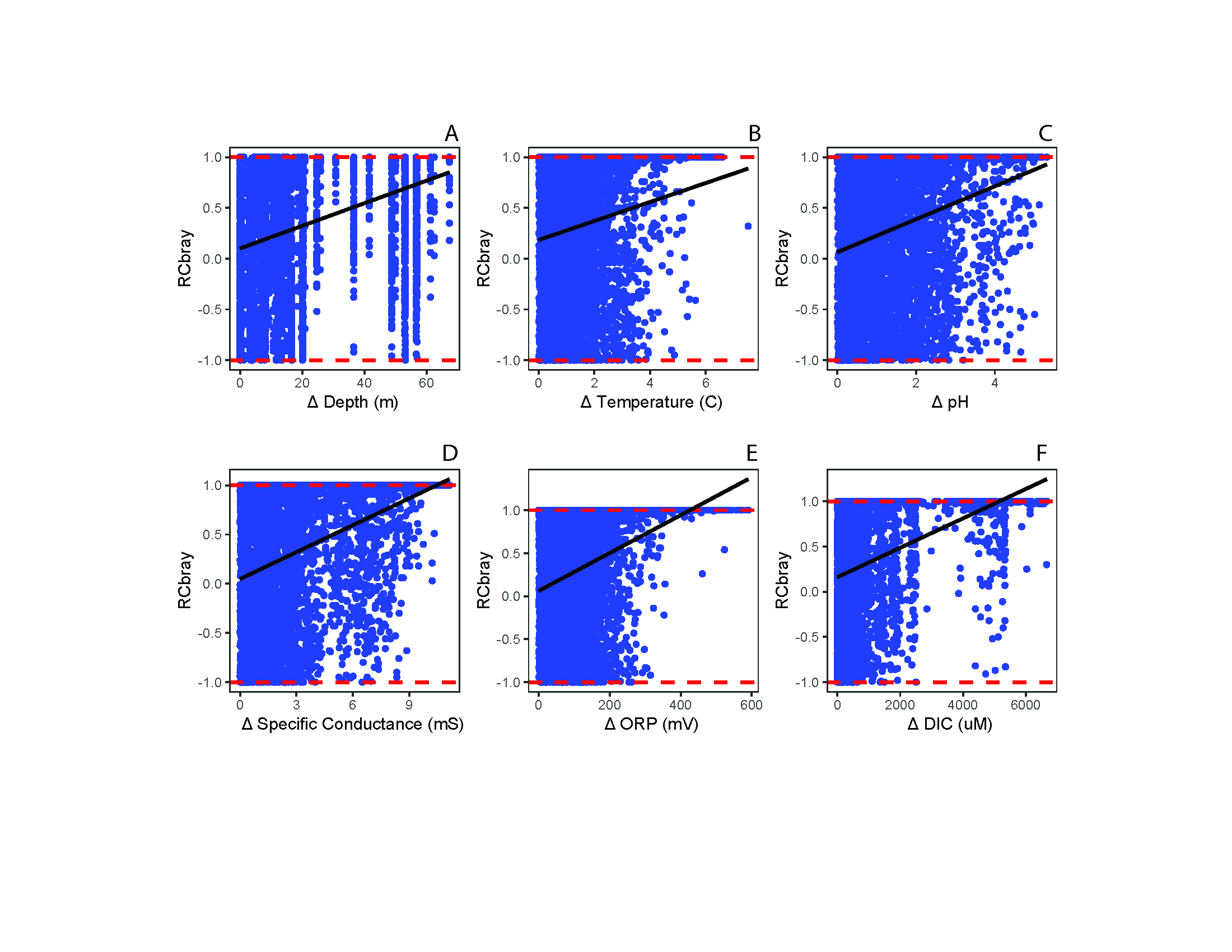

Supplement: FIG S3 [file msystems.00300-21-sf003.tif]
